# 3D Data Processing and Entropy Reduction for Reconstruction from Low-Resolution Spatial Coordinate Clouds in a Technical Vision System

**DOI:** 10.3390/e26080646

**Published:** 2024-07-30

**Authors:** Ivan Y. Alba Corpus, Wendy Flores-Fuentes, Oleg Sergiyenko, Julio C. Rodríguez-Quiñonez, Jesús E. Miranda-Vega, Wendy Garcia-González, José A. Núñez-López

**Affiliations:** 1 Facultad de Ingeniería, Universidad Autónoma de Baja California, Mexicali 21280, Mexico; ivan.alba@uabc.edu.mx (I.Y.A.C.); julio.rodriguez81@uabc.edu.mx (J.C.R.-Q.); wendy.garcia26@uabc.edu.mx (W.G.-G.); 2 IT de Mexicali, Tecnológico Nacional de México, Mexicali 21376, Mexico; elias.miranda@itmexicali.edu.mx; 3 Instituto de Ingeniería, Universidad Autónoma de Baja California, Mexicali 21100, Mexico; srgnk@uabc.edu.mx (O.S.); jose.nunez10@uabc.edu.mx (J.A.N.-L.)

**Keywords:** 3D reconstruction, spatial coordinate cloud, cloud registration

## Abstract

This paper proposes an advancement in the application of a Technical Vision System (TVS), which integrates a laser scanning mechanism with a single light sensor to measure 3D spatial coordinates. In this application, the system is used to scan and digitalize objects using a rotating table to explore the potential of the system for 3D scanning at reduced resolutions. The experiments undertaken searched for optimal scanning windows and used statistical data filtering techniques and regression models to find a method to generate a 3D scan that was still recognizable with the least amount of 3D points, balancing the number of points scanned and time, while at the same time reducing effects caused by the particularities of the TVS, such as noise and entropy in the form of natural distortion in the resulting scans. The evaluation of the experimentation results uses 3D point registration methods, joining multiple faces from the original volume scanned by the TVS and aligning it to the ground truth model point clouds, which are based on a commercial 3D camera to verify that the reconstructed 3D model retains substantial detail from the original object. This research finds it is possible to reconstruct sufficiently detailed 3D models obtained from the TVS, which contain coarsely scanned data or scans that initially lack high definition or are too noisy.

## 1. Introduction

The progress of 3D scanning technology has revolutionized the way we capture and replicate the physical world in digital form. From documentation of historical artifacts [1,2] to the precision required in industrial design, the applications of this technology are vast and varied. Of these systems, the versatility of laser scanning systems as remote sensing technology is the reason they are used across a wide array of fields. These systems are integral to various sectors, including healthcare, geospatial surveying, additive manufacturing, the mining industry, and reverse engineering, to highlight just a few. The application of laser scanning in the medical field enables precise mapping and modeling of physical forms, while in geospatial surveying, it facilitates the detailed assessment of land topographies and architectural structures. In the field of additive manufacturing, laser scanning contributes to the accuracy and fidelity of 3D-printed objects, and in mining, it aids in the volumetric analysis and planning of extraction sites. Reverse engineering processes benefit significantly from laser scanning by providing a means to recreate models of existing physical objects with high accuracy.

Multiple studies illustrate the influence of laser scanning technologies in both research and practical applications. For instance, ref. [3] explores the measurement of vegetation canopy structures using laser scanning, showcasing the method’s capability to capture detailed canopy data in situ without direct contact. The authors of [4] introduce innovative vegetation indices derived from terrestrial laser scanning, which effectively quantify the 3D spatial organization of plant communities. Another example is [5], which presents an analysis of a 3D laser tracking scanner system, focusing on its immunity to the effects of ambient sunlight and its geometric resolution. For a thorough exploration of the historical development and diverse applications of 3D laser scanners, ref. [6] offers an extensive overview, tracing the technology’s evolution and its impact across different industries. Over the years, the evolution of 3D scanning technology has led to the development of increasingly sophisticated devices that are adept at digitally capturing objects in their immediate surroundings, accurately registering even the smallest details and complex surfaces without the need for pre-defined reference points or markers. Additionally, many contemporary 3D laser scanners enhance their data collection capabilities by integrating GPS, providing further insights into the surfaces being scanned [7,8]. These technologies use light to amass substantial volumes of data—encompassing millions of discrete points—which are subsequently compiled into large point cloud files. This approach not only significantly improves the precision of digital representations but also broadens the scope for detailed examination and application of the gathered information. These devices have improved their underlying principles to not only become more efficient but also to capture highly accurate point clouds. The versatility of these 3D point clouds has led to their widespread application across various domains, including the inspection and mapping of structures [9] and landscapes [10], object recognition [11,12], and autonomous navigation [13], among others. Despite their utility, point clouds often exhibit minor variances in the coordinates of each scanned point, indicating that details finer than the scanner’s resolution may not be accurately captured. This limitation introduces a certain degree of imprecision to digital scanning, similar to the resolution constraints found in photographic scanners, thus establishing a limit to the proximity at which each laser track can accurately reflect the subject’s details.

In the domain of industrial applications, two primary types of scanners have emerged as leaders: contact and non-contact scanners. Contact scanners are known for their precision, while non-contact scanners boast quicker operation. The majority of non-contact 3D spatial coordinate measurement systems depend on optical sensors coupled with sophisticated signal processing techniques. These systems capture the surfaces of objects in discrete segments to ascertain their coordinates, facilitating the creation of a point cloud. This cloud can then be analyzed to accurately reconstruct an object’s shape and dimensions [14]. Such contactless technologies are primarily categorized into two main groups based on their operation: camera-based and laser-based. Additionally, they can be distinguished by their form, being either handheld or desktop devices. Handheld scanners often employ structured light technology, which projects a specific light pattern onto the target object. Dual cameras then capture and analyze the projected light patterns, discerning differences across various vision fields. Meanwhile, other camera-based scanners leverage an arranged array of sensors to gather 3D data from multiple angles around the object [15], enhancing the depth and accuracy of the data collected. Some devices enhance accuracy through the use of calibration patterns, enabling precise measurements from numerous perspectives around the object. In contrast, laser triangulation scanners use one or more laser beams directed at specific points on the object’s surface [16]. This method measures the distance between the scanner and these points, iteratively capturing spatial data to cover the entire area within the desired field of view. Certain laser scanners are equipped with level compensation mechanisms, designed to counterbalance any movements during the scanning process, thereby ensuring the integrity of the data collected. Moreover, specialized scanners might incorporate rotating platforms to adjust the positioning of the subject and the direction of light reception, allowing the 3D scanner to achieve measurements with enhanced precision [17]. The prevalence of optical sensor-based technologies derives from their non-invasive nature, eliminating the need for direct contact with the objects under study. Among their most valued attributes is the rapid rate at which measurements can be acquired. Nonetheless, notable limitations of these systems are their susceptibility to measurement inaccuracies caused by optical ambient noise and the complexities inherent in their design. Despite the advances in precision and accuracy, the data procured from 3D scanning processes frequently exhibit characteristics that can significantly undermine the quality of the resulting models. These imperfections manifest in various forms, including low resolution, noise, and the presence of outliers, each contributing to a reduction in the clarity and usability of the data. Moreover, while camera-based scanning technologies are known for producing data that may include a higher proportion of outliers [18], particularly under adverse lighting conditions or in environments with reduced visibility due to factors like rain or fog [19], laser-based scanners, though more precise, can be slower and may struggle with accurately detecting reflections from certain surfaces, potentially leading to additional outlier data. To address these problems, various methods for detecting and filtering outlier data in point clouds have been developed and applied across a range of fields, including bioengineering, reverse engineering, civil and archaeological engineering, as well as electronics and industrial automation. The implementation of point cloud outlier filtering techniques facilitates the creation and reconstruction of meshes and geometric models that closely resemble physical objects in appearance and dimensions. For instance, ref. [20] discusses several point cloud filtering methods, and ref. [21] have developed an algorithm that leverages surface variation factor segmentation, applying different filtering techniques to various sections of the point cloud for enhanced results across different regions. Additionally, ref. [22] explores the use of AI to design adaptive filters that consider the size and shape of the specific point cloud region being processed, further refining the accuracy and reliability of the resultant digital models. Extensive research on 3D scanning technologies and techniques was conducted prior to the experiments detailed in this paper, leading to the publication of a comprehensive review [23]. This review provides an in-depth analysis of various 3D scanning methods, their applications, and advancements in the subject.

This paper is structured as follows:

First, in the Materials and Methods section, the basic function of the TVS is explained and the procedure to scan objects using this system is described. An explanation of the methods used to reduce noise in the point clouds obtained from the TVS, as well as smoothing techniques and the algorithms used to compare the results are also included in this section. Second, the methodology and experimental results are presented, where details of the experimentation conducted, including the scanning of the original volumes, rotation of coordinates, reduction of outliers and entropy are described, as well as the results of these algorithms in the form of tables. In this section, a direct link to visualize the 3D point clouds results is included as well as flat images of the scanned faces. Third and last, the discussion and conclusions are presented, where the results of the experiments are summarized, and future possible work is discussed.

## 2. Materials and Methods

At the Autonomous University of Baja California, a method referred to as dynamic triangulation has been in use to measure spatial coordinates. This method involves adjusting the angles of the system’s laser emitter and receiver through motor control. The process allows the laser to target a specific area within the viewing field, reflecting off an object’s surface and being detected by a scanning aperture. This aperture rotates at a steady rate, with the objective of capturing the reflected laser light. Based on this principle, the Engineering Institute of the Autonomous University of Baja California has developed three prototypes of a “Technical Vision System” (TVS), designed to measure 3D coordinates within a given field of view. These prototypes use a single light sensor for gathering surface information from scanned objects, supporting a variety of applications. Uses for the system range from navigation and mapping in industrial settings [24], including pipelines [25], to structural monitoring and aiding in the medical field for the detection of spinal column issues. Some of the systems based on this principle use this technique in combination with cameras to adjust to the object’s position in real-time. Using this method, information on the energy distribution of the scanned object can also be obtained as well as information about the color of the material, alongside the angles of the actuators. With this information, the location in space of the object can be triangulated using basic trigonometry.

### 2.1. Technical Vision System Specifications and Object Scanning

**Figure 1:**
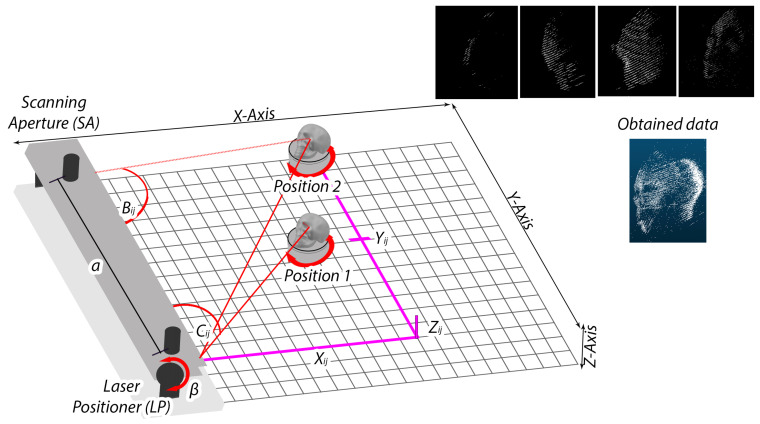
Point cloud creation in TVS2.

**Figure 2:**
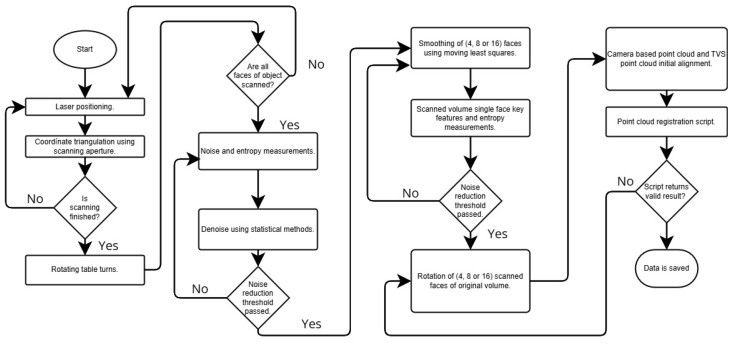
Flowchart for 3D data processing and entropy reduction for reconstruction.

TVS prototype number 2 (Figure 1), in particular, focuses on capturing the planar faces of objects to create point clouds. This system consists of three primary components: a laser positioner (LP), a scanning aperture (SA), and a positioning arm (PA). These elements work together using the principle of dynamic triangulation. This method measures the distances and angles of 3D points on the observed objects and converts them into rectangular coordinates to assemble the point clouds, while the system adjusts the angles between the system’s scanning aperture and the laser positioner to accurately measure the objects. A rotating base is used to obtain data from multiple faces of an object for later processing into a complete object, following the method described in Figure 2.

The better performance of TVS occurs at position 1 in its field of view for 3D points scanning. It has been previously determined, however, that the field of view was focused on a front view of the object. In this research, with the purpose to obtain multiple faces to integrate the whole volume, it was identified that in some table rotatory angles, the body presented laser occlusions due to protuberances of the object (as represented in Figure 1 with skull at position 1; with this table rotation angle the laser is occluded by the nose of the skull and can not be reached by the scanning aperture. Note that this occurs only for some of the 3D coordinates, not for all the 3D coordinates of the face view ), which motivated evaluation of measurements in position 2.

**Figure 3:**
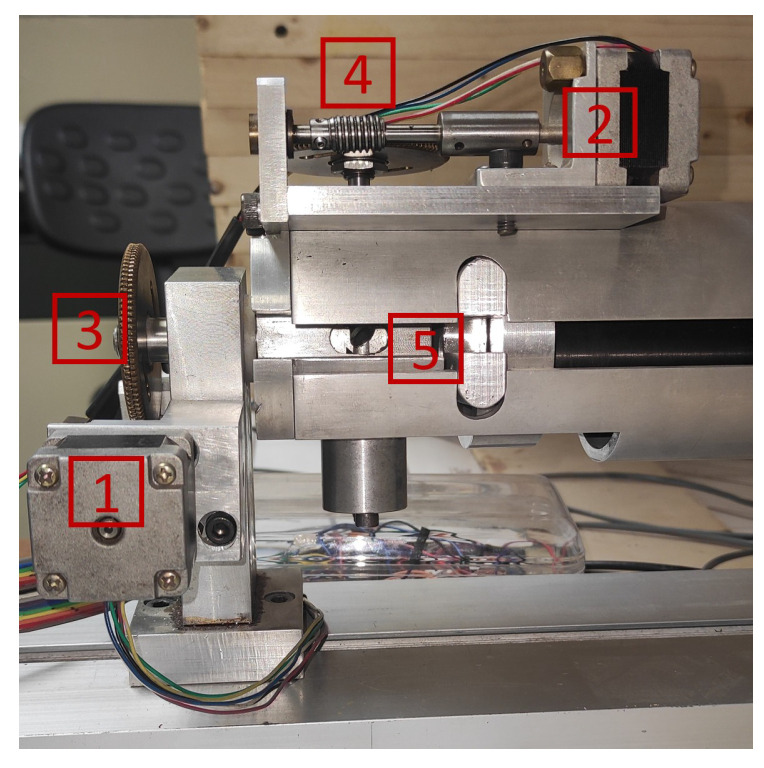
Laser positioner of TVS2.

**Figure 4:**
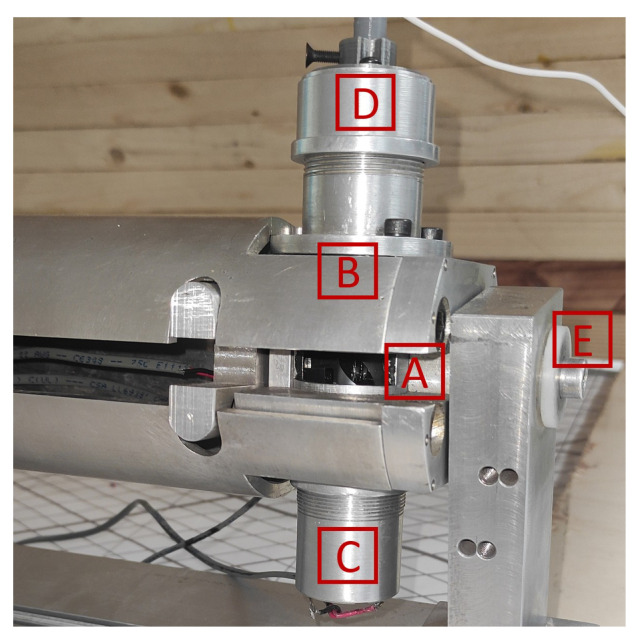
Scanning aperture of TVS2.

Figure 3 and Figure 4 illustrate the key components of the TVS prototype 2, detailing the construction and functionality of the system. Figure 3 focuses on the laser positioner’s elements, highlighting: (1) a stepper motor enabling rotation around its axis; (2) another stepper motor attached to a 45° mirror for directing the laser beam; (3) a gear-worm screw combination facilitating the rotation of the positioning arm; (4) a similar gear-worm mechanism for adjusting the 45° mirror’s position; and (5) the 45° mirror itself, which reflects the laser towards the target object.

Conversely, Figure 4 depicts the scanning aperture, comprising: (A) a 45° mirror for laser reflection; (B) lenses that focus the laser light; (C) a DC motor for mirror rotation; (D) a photosensor for detecting the laser light; and (E) Teflon bearings for smooth movement. The scanning aperture’s DC motor rotates freely, powered by a regulated voltage source to achieve the desired speed. However, due to this setup, the motor’s speed may vary, necessitating speed measurements at each revolution with an opto-coupler to maintain accuracy. The laser positioner employs a 10 mW red laser, aimed using stepper motors capable of 19,200 steps per revolution. The system’s photosensor, a phototransistor model BPW77N by VISHAY Semiconductor, captures the reflected laser light for further processing.

The scanning aperture acts as a receiver for the system. For the experiment, a specific scan window is programmed so that only 3D information of the object under measurement is obtained. Then, a rotatory base is used to scan a determined number of planar faces of the object. The scanning aperture then gathers data for each point, such as the energy distribution curve of each measured point, by reflecting the laser off the object onto a photosensor. The laser positioner, on the other hand, is responsible for directing the laser beam across different programmed points over the object in the defined scanning window, allowing the system to measure the reflection angles ($B_{ij}$) via the scanning aperture.

After capturing the data, they are stored in various files for further processing. The next step is to identify the peak of the signal for each point, determine when this peak signal occurs, and relate it to the position of the scanning aperture at that time. This step is applied to every point within the set scanning window, preparing the foundation for the detailed work of creating a point cloud. The next part of the process uses the angles of the laser positioner ($C_{ij}$) and the tilt (β angle) of the TVS itself. By applying basic trigonometry rules, shown in Equations (1)–(3), the (x,y,z) coordinates for each point are calculated. Where *a* is the distance between PL and SA. This process turns the collected data points into a structured point cloud.

(1) $$x_{ij}=a\frac{\sin B_{ij}\sin C_{ij}}{\sin\left(B_{ij}+C_{ij}\right)}$$

(2) $$y_{ij}=a\left(\frac{1}{2}-\frac{\cos Bij\sin C_{ij}}{\sin\left(B_{ij}+C_{ij}\right)}\right)$$

(3) $$z_{ij}=a\frac{\sin B_{ij}\sin C_{ij}\tanh\beta }{\sin\left(B_{ij}+C_{ij}\right)}$$

### 2.2. Noise and Outliers

In the scanning process, various factors can introduce noise and outliers into the data captured by this system. These irregularities are often due to problems such as the scanning aperture mirror speed not being constant, difficulties in accurately detecting the energy center of the signal during data processing, and the tilt angle of the system. These elements can complicate the accuracy of the data, making it challenging to maintain the integrity of the results. Noise and outliers can also obscure important details in the point clouds, requiring filtering to improve the results. While triangulation errors are more common along the X-axis, outliers can also appear on other axes, making it difficult to maintain clarity in the point clouds. Addressing these issues is crucial for refining the data and ensuring that the point clouds produced are as precise and representative of the scanned object as possible.

Outliers refer to data points that significantly differ in value from the majority of a dataset, being much higher or lower than the surrounding data. Within the context of point clouds, these outliers can negatively impact both the visualization of the point clouds and any further applications of the data. Their presence can distort the overall representation of the object, leading to inaccuracies in analysis or challenges in utilizing the data for modeling or other purposes.

### 2.3. Data Processing and Outlier Removal

For the majority of the outlier data obtained, statistical filters are applied on different axes of the point clouds obtained, depending on several factors; in particular, three methods are mentioned for this purpose:

#### 2.3.1. Interquartile Method

This method [26] applies to univariate data, which can be segmented into quartiles. The first quartile signifies that 25% of the data points fall below this value, the second quartile represents the median of the dataset, and the third quartile shows that 25% of the data points exceed that value. The interquartile method is employed to determine the data range that should be kept, given that:

(4) $$\left[Q_{1}-K\left(Q_{3}-Q_{1}\right),Q_{3}+K\left(Q_{3}-Q_{1}\right)\right]$$

where *K* is a given constant and has a non-negative value, which can be used to control the amount of data to be detected as outliers, while $Q_{1}$ and $Q_{3}$ correspond to the quartiles used as upper and lower thresholds for the data. Values outside the threshold defined by Equation (4) are considered outliers.

#### 2.3.2. Modified Thompson Tau Method

The modified Thompson Tau method [27] is an alternative strategy for identifying outliers within a dataset. Here, the approach requires dealing with a single variable dataset, assessing potential outliers one at a time and removing those found to be beyond two standard deviations from the dataset’s mean.

To apply this technique, one must first determine the mean absolute deviation δi, followed by calculation of the Tau threshold τ:

(5) $$\tau =\frac{t_{\alpha /2}\left(n-1\right)}{\sqrt{n}\sqrt{n-2+{\left(t_{\alpha /2}\right)}^{2}}}$$

where $t_{\alpha /2}$ is the inverse cumulative distribution function and *n* is the size of the dataset. In this way, a value is determined to be an outlier if it is found that δi > τ∗S, where *S* is the standard deviation.

#### 2.3.3. Chi^2^ Distribution Quantiles

In this method [28], for each sample of dimension *d*, the Mahalanobis distance is determined given that:

(6) $$Di=\left(X_{i}-\bar{X}\right)*\sum^{-1}*{\left(X_{i}-\bar{X}\right)}^{\prime }$$

where all values of Di exceeding the calculated critical value are detected.

#### 2.3.4. Smoothing of the Point Cloud

After the outlier processing, the Moving Least Squares (MLS) [29] algorithm is used. This technique is based on weighted linear regression. The MLS method is particularly effective for reconstructing surfaces from point sets, and is often employed to create 3D surfaces from point clouds through downsampling or upsampling. Its utility is notable in handling data that are irregularly spaced or contain noise, as it provides a flexible fitting approach that adapts to the local data structure.

(7) $$S=\sum_{i=1}^{n}w_{i}\;\cdot \;{\left(y_{i}-f\left(x_{i}\right)\right)}^{2}$$

where *S* is the sum that needs to be minimized, *n* is the number of data points, and $w_{i}$ represents the weight for the *i*-th data point, often a function of the distance to emphasize points closer to the point of interest. $y_{i}$ and $x_{i}$ are the coordinates of the *i*-th data point and $f(x_{i})$ is the value of the approximation function at $x_{i}$.

### 2.4. Point Cloud Registration

Used in computer vision, robotics, and 3D reconstruction, point cloud registration is the process of aligning multiple point clouds, each captured from different positions within the same environment, into a unified coordinate framework. This process results in a consolidated dataset that accurately represents the scene being surveyed [30]. For this experiment, two similar point cloud registration algorithms were used, the Iterative Closest Point (ICP) for local registration and the Random Sample Consensus (RANSAC) for global registration via the Open3D library [31].

#### 2.4.1. Iterative Closest Point Registration (ICP)

This process involves aligning two or more point clouds by identifying a spatial transformation, which could involve scaling, rotation, or translation and reduces the disparities between them. The ultimate goal is to combine these multiple datasets into a cohesive, globally consistent model or coordinate framework. The process begins with two point clouds and an initial transformation that approximately positions the source point cloud in alignment with the target point cloud. The result is a transformation that closely and accurately aligns the two point clouds.

(8) $$E\left(R,t\right)=\sum_{i=1}^{N}{∥\left(R\;\cdot \;p_{i}+t\right)-q_{i}∥}^{2}$$

where *R* is the rotation matrix, *t* is the translation vector, $p_{i}$ are the points from the source dataset, $q_{i}$ are the corresponding closest points in the target dataset, and *N* is the number of point pairs.

#### 2.4.2. Random Sample Consensus Registration (RANSAC)

Another category of registration techniques, referred to as global registration, encompasses algorithms that do not necessitate an initial alignment to start. Typically, yielding less precise alignments, this method utilizes RANSAC [32] for the initial alignment of the two point clouds, and then ICP is used for the comparison.

### 2.5. Ground Truth

For comparison with the original objects’ dimensions in the experiment, an Intel RealSense camera was used to measure the proportions of the objects. This camera integrates an advanced stereoscopic vision system, capturing depth information of objects in their field of view in addition to traditional 2D images.

### 2.6. Reconstruction

Reconstruction uses the screened Poisson algorithm [33], which is used to create a smooth continuous surface representing the scanned object or scene from a point cloud. It is based on the Poisson equation, where in a three-dimensional Cartesian coordinate system, it takes the form:

(9) $$\left(\frac{\partial^{2}}{\partial x^{2}}+\frac{\partial^{2}}{\partial y^{2}}+\frac{\partial^{2}}{\partial z^{2}}\right)\varphi \left(x,y,z\right)=f\left(x,y,z\right)$$

where φ and *f* are real or complex functions.

## 3. Methodology and Experimental Results

The next section outlines the method used to perform several 3D scans on different objects, using two configurations. It describes how a 3D scan performed by the TVS 2 on different sides of an object is compared to scans from an Intel RealSense camera, using a point cloud registration algorithm for the comparison. The results of this process are then presented.

### 3.1. Design of Experiment

The main experiment involved conducting 3D scans to evaluate the performance of the Technical Vision System (TVS) under various scanning resolutions and window configurations. The objective was to compare the efficiency of 3D scans produced by the TVS 2 across multiple faces of an object utilizing both a standard scanning setup and a scan made using an Intel RealSense camera. This comparison focused on assessing the ability of the TVS 2 to maintain sufficient detail and accuracy against scans from an Intel RealSense camera, to find a way to minimize time and scan windows when utilizing the TVS system, and then employing a point cloud registration algorithm for analysis.

Factors considered during these experiments included the ambient lighting conditions in the laboratory, adjustments to the scanning aperture’s (SA) mirror speed to test for consistency and noise reduction, and the spatial arrangement between the TVS 2. The following Table 1 summarizes the experimentation factors ranges and their levels:

The experiment was conducted using a rotating base (for object orientation related to the front of TVS) controlled by a stepper motor and a reduction gear for precise manipulation. The chosen scanned objects for the experiment included letter and number-shaped figures and a polystyrene foam skull. The number of faces to be scanned for each object was defined, as well as the scanning resolution—determined by the number of points to be obtained—and the number of light sensor readings per scanned point. The object on the rotating base was positioned in two different locations in front of the TVS. These settings can be appreciated in Table 2.

The TVS system scans each pre-defined face of the object and positions the laser at specific points defined as part of the scan window and resolution settings. It records the position data of each TVS component for later compilation, along with the voltage readings from the system’s photosensor, which employs a basic common-collector arrangement with a resistor. Once data collection from the scan window is complete, the rotating base moves a preset number of degrees, generating a file for each point defined within the scan window.

After the scanning process, the data are processed on a personal computer. Using Equations (1)–(3), point clouds for each face of the selected objects are generated. Due to the TVS design, these point clouds tend to include noise and outliers. Therefore, various statistical filters are employed to minimize these data anomalies. An example of this reduction is illustrated in Table 3, showcasing the standard deviation reduction on the X-axis for one face of a polystyrene object in the form of the letter “A”. The noise levels and outlier characteristics observed for the letter ‘A’ were representative of all other foam objects examined in this study. The data undergo smoothing through the Moving Least Squares (MLS) method, resulting in a more accurate and smoother representation of the object surface.

Following the data acquisition for each face, the point clouds from the captured faces are translated, using the center of the rotating table as the new center for the point cloud. This involves defining two points, *P* and *O*, which represent spatial coordinates in a three-dimensional space as follows: $P=[P_{1}P_{2}P_{3}]$ and $O=[O_{1}O_{2}O_{3}]$.

The distance vector between these two points can be calculated as follows: $\vec{PO}=O-P$. The magnitude of both distance vectors is computed, and the vector to be translated is determined. Subsequently, a transformation matrix is used to perform the point cloud translation:

(10) $$P^{\prime }=\left[\begin{array}{c} P_{1}^{\prime }\\ P_{2}^{\prime }\\ P_{2}^{\prime }\\ 1 \end{array}\right]=T_{\alpha }P=\left[\begin{array}{cccc} 1 & 0 & 0 & V_{\alpha }\\ 0 & 1 & 0 & V_{\alpha }\\ 0 & 0 & 1 & V_{\alpha }\\ 0 & 0 & 0 & 1 \end{array}\right]\left[\begin{array}{c} P_{1}^{\prime }\\ P_{2}^{\prime }\\ P_{2}^{\prime }\\ 1 \end{array}\right]=P+V_{\alpha }$$

The data are then rotated using simple rotation matrices around the Z-axis.

(11) $$\left[\begin{array}{ccc} cos\theta & -sin\theta & 0\\ sin\theta & cos\theta & 0\\ 0 & 0 & 1 \end{array}\right]\left[\begin{array}{c} X_{i}\\ Y_{i}\\ Z_{i}\\ 1 \end{array}\right]=\left[\begin{array}{c} X_{f}\\ Y_{f}\\ Z_{f}\\ 1 \end{array}\right]$$

Entropy measurements are used to provide a quantitative assessment of the randomness or disorder within the point cloud data generated by the Technical Vision System (TVS). Entropy, a concept from information theory, offers a straightforward metric to assess the variability in the data, which can be indicative of noise levels or scanning inconsistencies. The approximate entropy and Shannon entropy measures can be seen in Table 4 and Table 5.

**Figure 5:**
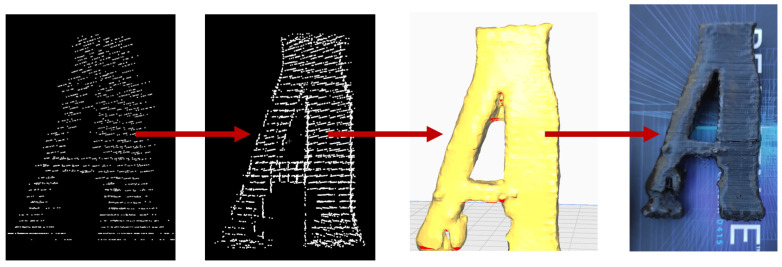
From point cloud to 3D print.

After the rotation of the points, a “complete” cloud is generated and a 3D solid is created via the screened Poisson algorithm. This reproduction is ready to edit or 3D print (Figure 5).

### 3.2. Results of the Experiment

After obtaining a “complete” point cloud and mesh from all faces of the object, the ICP algorithm was used to compare the similarities between the point cloud obtained from the RealSense camera and the TVS 2. This section describes the process and results.

#### 3.2.1. Evaluation of Similarities

Our examination focused on specific measurements within recognizable features of the polystyrene objects, skull, letter “A” and number “6”, made from the same material. It was observed that distances critical to the identification of the objects, such as the gap between the eyes and nose on the skull, or the space within the letter “A”, consistently fell within the tolerances allowed by the scanning window, as seen in Table 6. This outcome suggests that, due to the discrete nature of the scanning window in the TVS, perfect replication of corner measurements would require a continuous field of view. Moreover, a cloud registration algorithm was used to facilitate feature matching for global registration by first simplifying the camera-based point cloud for an effective comparison with the TVS-generated cloud, and then performing a manual alignment between the two point clouds.

**Figure 6:**
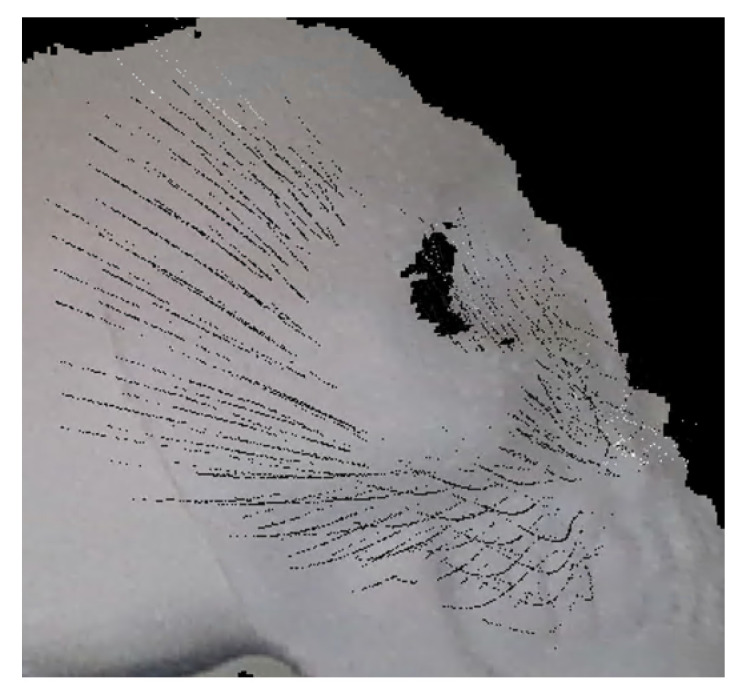
Skull face scanned by TVS.

It was observed that the obtained data from the TVS were not able to obtain a fitness score greater than 0. This was attributed to an excessive amount of noise in the data prior to processing of the point clouds. It was also observed that the simpler geometric objects, such as the letter “A”, reached volumetric fitness scores as high as 0.96, as seen in Table 7 as well as other results. In contrast, the more intricate features of the polystyrene skull posed a challenge, resulting in less precise scans with fitness scores peaking at 0.88 in volumetric form and 0.50 on some of the scanned faces, which can be seen in Table 8, accompanied by representative images of the scanned faces of the skull in Table 9, despite certain faces meeting the criteria for key comparison measurements. This effect was attributed to deformations made by the reflected laser light not reaching the scanning aperture when reaching a surface with a significant curvature, such as the representation of the zygomatic bones on the skull, leading to the algorithm not being able to recognize features in some cases. An example of this deformation can be seen in Figure 6, where part of the TVS-based cloud was aligned with the camera-based point cloud to compare the similarities. It can be observed how the lines of the TVS scanning, represented in black, follow the basic structure of the skull, being interrupted in the area representing the zygomatic bones in the object.

#### 3.2.2. Comparison with Unfiltered Data

The benefits of post-processing when using this system can be seen by comparing the filtered data with their unfiltered counterpart. In particular, for some of the unfiltered faces of the polystyrene skull, the presence of too much noise in the data made it impossible to make accurate key measurements, while the global registration algorithms were ineffective, failing to produce a score greater than 0. This highlights the importance of the statistical filtering methods used and data processing in mitigating inaccuracies in the TVS, thus enhancing the clarity and usability of the generated point clouds. The results of the filtered data in regard to entropy reduction can be seen in Table 4 for the “Approximate Entropy Method” and Table 5 for the “Shannon Entropy Method”. The key measurements from some of the volumes can be seen in Table 6; fitness scores for the volumes and faces can be seen in Table 10 for Letter A and Number 6 faces, in position 1. Note that evaluation in position 2 was not required due to the objects being mainly flat and no occlusion of the laser was produced due to the object face flatness. As shown in Table 8, for skull faces at position 1 and position 2, due to the skull body not being flat and causing occlusion of the laser in some protuberant points (for example, the nose not allowing you to see the cheek), when positioned on position 1, it was necessary to evaluate the measurements at position 2 to avoid occlusion of the laser for the scanning aperture. Finally, Table 7 presents information for the Letter A, Number 6, and Skull volumes. A live 3D view [34] link has also been provided.

#### 3.2.3. Limitations and Uncertainties

The precision of the measurements and the effectiveness of the registration algorithm are bound by the resolution and fidelity of the scanning process, which is influenced by the material properties of the objects and the scanning environment, as well as the age of the system and the behavior of the laser scanning systems. Furthermore, the discrete nature of the scanning window introduces an unavoidable element of approximation in capturing the fine details of complex geometries. Such factors contribute to the variability in fitness scores and highlight the challenges of achieving high fidelity in 3D scanning and model comparison.

## 4. Discussion and Conclusions

In this study, the performance of the Technical Vision System (TVS) across various scanning resolutions and window configurations was explored with the aim of obtaining a 3D solid faithful to the original scanned object, based on the TVS scanned faces while using a rotating table, focusing on the accuracy of 3D scans in capturing key features of different polystyrene objects. The original aim of the study was to investigate the inconsistencies observed in the quality of the 3D scans produced by the system when scanning objects located at different positions within the system’s field of view. Significant challenges were posed by noise and other particularities of the TVS, such as the speed of the scanning aperture, which had to be addressed by different techniques to mitigate these problems. Among the techniques used, the statistical filters used for data processing and the MLS algorithm greatly increased the fidelity of the 3D models, as well as the fitness rate of the comparison between the cloud points. The study has been successful in identifying areas of the field of view that appear to produce better results in obtaining 3D solids that are comparable to the original in their key features and maintain a similar fitness score to the original object while reducing the approximate entropy of the data. The experiments were performed in a controlled environment due to the original and main purpose of the research being to scan and digitally reconstruct objects using a rotating table. This setup allowed for obtaining multiple scans from different faces of an object for inverse engineering. However, after recognizing the potential of the TVS system for 3D scanning at reduced resolutions and optimal scanning windows, we explored the use of statistical data filtering techniques and regression models. These methods enable the generation of a 3D scan that remains recognizable with the least amount of 3D points. This approach balances the need for accurate object representation with the efficiency required for practical applications, such as robotics navigation. By reducing the number of points needed for a reliable scan, a scanning system like the TVS could more quickly process and understand the scene in the robot’s field of view, supported by deep learning classification algorithms. Potential application in robotics navigation involves balancing the number of points scanned with the time required to comprehend the scene, optimizing both accuracy and processing efficiency.

In this study, it was possible to obtain recognizable solid figures from low-quality, high-noise point clouds created using a Technical Vision System, as well as reducing the entropy in the data by up to 57%. The data obtained from the system represented multiple faces of a rotated object; each face was filtered using statistical methods, such as modified Thompson Tau and chi-square distribution quantiles. The data were then smoothed using the Moving Least Squares algorithm and then rotated to be assembled in a single point cloud representative of the original volume. To validate that the results from the algorithms were accurate, the data were compared to point clouds from an Intel Realsense camera using point cloud registration. The point clouds were compared to each scanned face by the TVS as well as the rotated representations. These results are shown in Table 8 for objects’ faces and Table 7 for volumes. The results show that for each face the maximum fitness score obtained was 50.6%, and once the faces were rotated and assembled in a single point cloud representation of the original volume, the maximum fitness score obtained was 96%. It was also shown that the first position selected for the field of view of the system was the optimum place to scan objects for this particular system.

However, the limitations of our experimental setup, including the choice of object materials and the controlled laboratory environment, suggest the need for further studies to assess the applicability of these techniques in varied scanning contexts. Future research should also consider the integration of alternative algorithms and the exploration of adaptive scanning configurations to address the nuanced challenges presented by different objects’ geometrical complexities. The experimental findings also confirmed that the quality of the scans varied significantly depending on the position, with Position 2 consistently yielding inferior results compared to Position 1. This variation can be attributed to factors such as the angle of incidence of the laser, the distance from the scanner, or potential obstructions that affect the scanner’s line of sight at different positions.

Ultimately, this study contributes to the evolution of the TVS, adding the capability to obtain complete 3D reconstruction of objects, while also offering insights into the optimization of the scanning processes and the potential for enhanced model reconstruction. By continuing to refine these methods, the intricacies of digital modeling can be integrated with the capabilities of the system, paving the way for broader applications in industrial design, heritage conservation, and beyond.

The findings of this work clearly indicate that Position 1 consistently delivers higher-quality scans compared to Position 2, highlighting the necessity of optimal scanner placement to ensure data accuracy. To address the challenges posed by less-favorable scanning positions, future development of the TVS should, therefore, focus on exploring adaptive scanning strategies that can mitigate the effects of sub-optimal positioning and increase the flexibility of the system. The use of more advanced data filtering and enhancement techniques will also be useful in improving the fidelity of 3D scans across varied operational environments, as well as in gaining the ability to compensate for positional disadvantages through more advanced data processing techniques. Additionally, ongoing improvements in the system’s design, operation, and hardware could further stabilize scanning outcomes, ensuring more consistent quality across different operational settings.

## Figures and Tables

**Table 1:** Experimentation factors.

Factor	Level/Range	Δ
Laboratory Light	OFF (<1 Lux)	-
Photosensor Voltage	3.3 V	-
Amplifier Resistance	830 KΩ	-
SA Rotating Speed	300 rpm	-
Depth Scanning Distance *x*	55–85 cm	35 cm

**Table 2:** Experiment settings for scanned foam objects.

Object	Rotating Table Angle Step	Obtained Faces	Position of Rotating Table in TVS (x,y,z) in cm	Scanning Window *y* × *z*
Letter A	90.0°	4	POS1 (33,30,10)	13° × 15°
Number 6	90.0°	4	POS1 (33,30,10)	13° × 15°
Skull	90.0°	4	POS1 (33,30,10) and, POS2 (21,23,10)	13° × 15°
Skull	45.0°	8	POS1 (33,30,10) and, POS2 (21,23,10)	13° × 15°
Skull	22.5°	16	POS1 (33,30,10) and, POS2 (21,23,10)	13° × 15°

**Table 3:** Standard deviation of face of foam letter A.

Method/Dataset	Quantity of 3D Points	Standard Deviation
Original	18,462	1.1100
Interquartile	9232	0.2350
Thompson Tau α = 0.01	17,239	0.5475
Thompson Tau α = 0.1	14,026	0.3751
Chi^2^ 97.5%	18,116	0.6900
Chi^2^ 90%	17,916	0.64200

**Table 4:** Approximate entropy calculated for each volume.

Object	Position	Rotating Table Angle Step	Obtained Faces	Approximate Entropy before Filtering	Approximate Entropy after Filtering
Letter A	Pos1	90.0°	4	0.384	0.244
Number 6	Pos1	90.0°	4	0.449	0.252
Skull	Pos1	45.0°	8	0.434	0.295
Skull	Pos1	90.0°	4	0.357	0.267
Skull	Pos1	22.5°	16	1.155	0.699
Skull	Pos2	45.0°	8	0.591	0.362
Skull	Pos2	90.0°	4	0.688	0.340
Skull	Pos2	22.5°	16	1.391	0.591

**Table 5:** Shannon entropy calculated for each volume.

Object	Position	Rotating Table Angle Step	Obtained Faces	Shannon Entropy before Filtering	Shannon Entropy after Filtering
Letter A	Pos1	90.0°	4	3.515	2.984
Number 6	Pos1	90.0°	4	3.007	2.878
Skull	Pos1	45.0°	8	4.365	3.899
Skull	Pos1	90.0°	4	3.850	3.162
Skull	Pos1	22.5°	16	4.835	4.023
Skull	Pos2	45.0°	8	4.871	3.846
Skull	Pos2	90.0°	4	3.576	3.104
Skull	Pos2	22.5°	16	3.777	3.255

**Table 6:** Measurement comparison of objects scanned in both locations from the TVS coordinate reference.

Object	Position	Measured Section	Target (cm)	TVS Scanned (cm)
Letter A	POS1	Bottom to top	15.8	16.0
Letter A	POS1	Central aperture (vertical)	4.2	4.5
Number 6	POS1	Bottom to top	9.6	9.7
Number 6	POS1	Central aperture (vertical)	2.2	2.2
Skull	POS1	Center of eye to nose	3.5	3.6
Skull	POS1	Jaw to top	13.8	14.0
Skull	POS2	Center of eye to nose	3.5	3.7
Skull	POS2	Jaw to top	13.8	14.3

**Table 7:** Fitness scores of volumes. Click over each image for better visualization on another window.

Object	Position	Rotation	Fitness Score	Flat 3D Image	External 3D View
Letter A	Pos1	90.0°	0.961	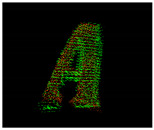	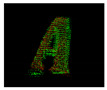 Click for 3D View
Number 6	Pos1	90.0°	0.919	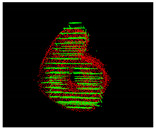	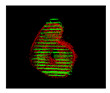 Click for 3D View
Skull	Pos1	90.0°	0.219	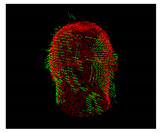	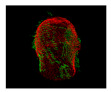 Click for 3D View
Skull	Pos1	45.0°	0.853	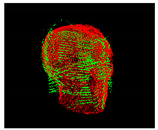	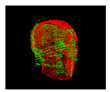 Click for 3D View
Skull	Pos1	22.5°	0.883	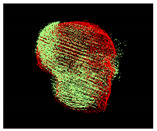	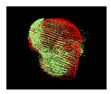 Click for 3D View
Skull	Pos2	90.0°	0.118	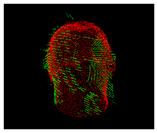	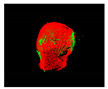 Click for 3D View
Skull	Pos2	45.0°	0.319	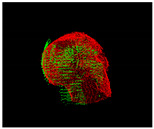	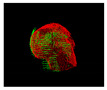 Click for 3D View
Skull	Pos2	22.5°	0.716	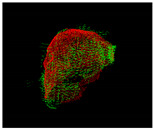	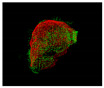 Click for 3D View

**Table 8:** Fitness scores of faces normalized from a maximum of 50.6% and minimum of 0%.

Object Foam Skull			
Position 1		Position 2	
Rotation	Maximum Fitness Score	Rotation	Maximum Fitness Score
Rot1of16	0.543	Rot1of16	0.260
Rot2of16	0.943	Rot2of16	0.202
Rot3of16	1.000	Rot3of16	0.159
Rot4of16	0.624	Rot4of16	0.00
Rot5of16	0.604	Rot5of16	0.481
Rot6of16	0.339	Rot6of16	0.159
Rot7of16	0.000	Rot7of16	0.000
Rot8of16	0.000	Rot8of16	0.000
Rot9of16	0.000	Rot9of16	0.000
Rot10of16	0.513	Rot10of16	0.000
Rot11of16	0.549	Rot11of16	0.000
Rot12of16	0.381	Rot12of16	0.149
Rot13of16	0.876	Rot13of16	0.206
Rot14of16	0.567	Rot14of16	0.240
Rot15of16	0.000	Rot15of16	0.503
Rot16of16	0.583	Rot16of16	0.381
Rot1of8	0.523	Rot1of8	0.130
Rot2of8	0.765	Rot2of8	0.210
Rot3of8	0.824	Rot3of8	0.015
Rot4of8	0.098	Rot4of8	0.220
Rot5of8	0.448	Rot5of8	0.080
Rot6of8	0.757	Rot6of8	0.402
Rot7of8	0.947	Rot7of8	0.341
Rot8of8	0.662	Rot8of8	0.543
Rot1of4	0.543	Rot1of4	0.341
Rot2of4	0.684	Rot2of4	0.143
Rot3of4	NA	Rot3of4	0.108
Rot4of4	0.583	Rot4of4	0.106

**Table 9:** Visual representation of objects’ faces used to calculate the fitness scores after spatial coordinate cloud processing. Red points correspond to the target, green points correspond to TVS measurements. Click over each image for better visualization on another window.

Object Foam Letter A					
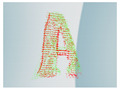 LetterARot1of4	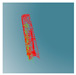 LetterARot2of4	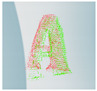 LetterARot3of4	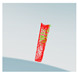 LetterARot4of4	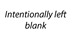 Intentionally left blank.	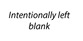 Intentionally left blank.
Object Foam Number 6					
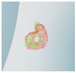 Number6Rot1of4	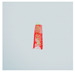 Number6Rot2of4	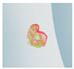 Number6Rot3of4	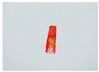 Number6Rot4of4	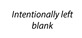 Intentionally left blank.	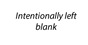 Intentionally left blank.
Object Foam skull					
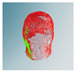 SkullPOS1Rot1of16	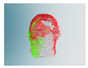 SkullPOS1Rot2of16	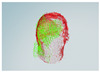 SkullPOS1Rot3of16	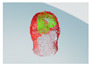 SkullPOS1Rot4of16	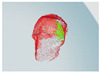 SkullPOS1Rot5of16	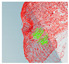 SkullPOS1Rot6of16
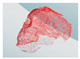 SkullPOS1Rot7of16	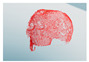 SkullPOS1Rot8of16	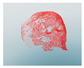 SkullPOS1Rot9of16	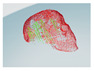 SkullPOS1Rot10of16	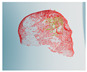 SkullPOS1Rot11of16	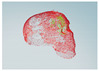 SkullPOS1Rot12of16
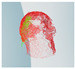 SkullPOS1Rot13of16	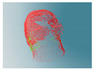 SkullPOS1Rot14of16	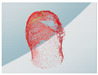 SkullPOS1Rot15of16	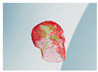 SkullPOS1Rot16of16	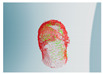 SkullPOS1Rot1of8	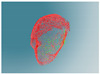 SkullPOS1Rot2of8
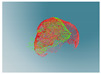 SkullPOS1Rot3of8	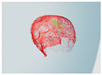 SkullPOS1Rot4of8	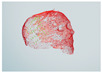 SkullPOS1Rot5of8	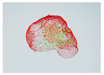 SkullPOS1Rot6of8	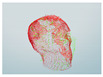 SkullPOS1Rot7of8	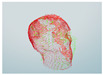 SkullPOS1Rot8of8
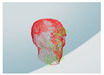 SkullPOS1Rot1of4	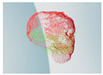 SkullPOS1Rot2of4	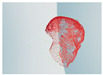 SkullPOS1Rot3of4	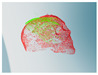 SkullPOS1Rot4of4	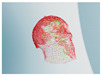 SkullPOS2Rot1of16	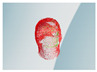 SkullPOS2Rot2of16
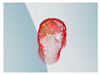 SkullPOS2Rot3of16	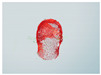 SkullPOS2Rot4of16	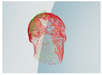 SkullPOS2Rot5of16	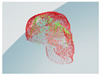 SkullPOS2Rot6of16	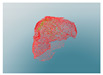 SkullPOS2Rot7of16	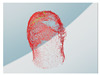 SkullPOS2Rot8of16
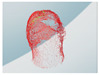 SkullPOS2Rot9of16	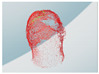 SkullPOS2Rot10of16	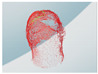 SkullPOS2Rot11of16	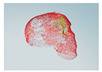 SkullPOS2Rot12of16	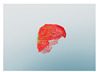 SkullPOS2Rot13of16	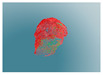 SkullPOS2Rot14of16
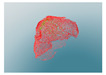 SkullPOS2Rot15of16	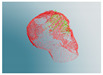 SkullPOS2Rot16of16	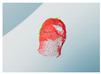 SkullPOS2Rot1of8	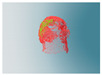 SkullPOS2Rot2of8	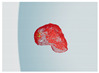 SkullPOS2Rot3of8	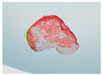 SkullPOS2Rot4of8
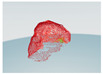 SkullPOS2Rot5of8	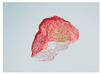 SkullPOS2Rot6of8	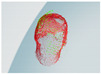 SkullPOS2Rot7of8	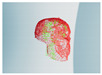 SkullPOS2Rot8of8	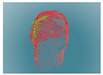 SkullPOS2Rot1of4	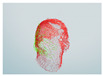 SkullPOS2Rot2of4
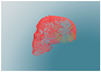 SkullPOS2Rot3of4	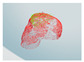 SkullPOS2Rot4of4	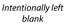 Intentionally left blank.	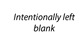 Intentionally left blank.	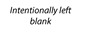 Intentionally left blank.	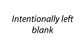 Intentionally left blank.

**Table 10:** Fitness score of faces of foam Letter A and Number 6 and at POS1.

Object			
Letter A at Pos1		Number 6 at Pos1	
Rotation	Maximum Fitness Score	Rotation	Maximum Fitness Score
Rot1of4	0.913	Rot1of4	0.750
Rot2of4	0.343	Rot2of4	0.263
Rot3of4	0.887	Rot3of4	0.765
Rot4of4	0.440	Rot4of4	0.360

## Data Availability

The point cloud data are available at https://uabc.appliedphysics.work/datapointclouds2024/MDPIData.zip (accessed on 17 July 2024).

## Abbreviations

The following abbreviations are used in this manuscript:

TVS	Technical Vision System
LP	Laser positioner
SA	Scanning aperture
MLS	Moving Least Squares
CONAHCYT	Consejo Nacional de Humanidades, Ciencias y Tecnologías
